# Testing Taxon Tenacity of Tortoises: evidence for a geographical selection gradient at a secondary contact zone

**DOI:** 10.1002/ece3.1500

**Published:** 2015-04-30

**Authors:** Taylor Edwards, Kristin H Berry, Richard D Inman, Todd C Esque, Kenneth E Nussear, Cristina A Jones, Melanie Culver

**Affiliations:** 1 School of Natural Resources and the Environment, The University of ArizonaTucson, Arizona, 85721; 2 University of Arizona Genetics Core, University of Arizona1657 E. Helen Street, Tucson, Arizona, 85721; 3 Western Ecological Research Center, U.S. Geological SurveyFalcon Business Park, 21803 Cactus Avenue, Suite F, Riverside, California, 92518; 4 Western Ecological Research Center, U.S. Geological Survey160 North Stephanie St., Henderson, Nevada, 89074; 5 Arizona Game and Fish Department, Nongame Wildlife BranchPhoenix, Arizona, 85086; 6 Arizona Cooperative Fish and Wildlife Research Unit, U.S. Geological Survey, University of ArizonaTucson, Arizona, 85721

**Keywords:** Cline, *Gopherus*, hybrid zone, Mojave Desert, secondary contact, Sonoran Desert

## Abstract

We examined a secondary contact zone between two species of desert tortoise, *Gopherus agassizii* and *G. morafkai*. The taxa were isolated from a common ancestor during the formation of the Colorado River (4–8 mya) and are a classic example of allopatric speciation. However, an anomalous population of *G. agassizii* comes into secondary contact with *G. morafkai* east of the Colorado River in the Black Mountains of Arizona and provides an opportunity to examine reinforcement of species' boundaries under natural conditions. We sampled 234 tortoises representing *G. agassizii* in California (*n* - 103), *G. morafkai* in Arizona (*n* - 78), and 53 individuals of undetermined assignment in the contact zone including and surrounding the Black Mountains. We genotyped individuals for 25 STR loci and determined maternal lineage using mtDNA sequence data. We performed multilocus genetic clustering analyses and used multiple statistical methods to detect levels of hybridization. We tested hypotheses about habitat use between *G. agassizii* and *G. morafkai* in the region where they co-occur using habitat suitability models. *Gopherus agassizii* and *G. morafkai* maintain independent taxonomic identities likely due to ecological niche partitioning, and the maintenance of the hybrid zone is best described by a geographical selection gradient model.

## Introduction

Exploring the relative importance of isolation and gene flow in the process of speciation is made possible when predictable patterns of divergence have occurred, such as regions where sister taxa come into secondary contact. When species diverge in allopatry, speciation may be incomplete because reinforcement is lacking and reproductive isolating mechanisms may not fully develop. Thus, hybridization may occur if the genetically distinct populations come into contact. Multiple models are used to describe hybrid zones where the amount of hybridization is dependent on factors such as dispersal ability, environment, and selection (Harrison 1993; Arnold 1997). Hybrid zones often are observed at ecotones between two distinct habitats (Harrison 1993; Arnold 1997) where exogenous selection may drive the amount of hybridization. Where hybrid zones are environment and dispersal dependent, a cline may be observed (Endler 1977) and cline width can be suggestive of the strength of selection (Smith et al. 2013).

The presence of a secondary contact zone between two species of desert tortoise (*Gopherus agassizii* and *G. morafkai*) in northwestern Arizona provides a natural experiment for testing the tenacity of these two taxa (Hewitt 1988). *Gopherus agassizii* (Agassiz's desert tortoise) and *G. morafkai* (Morafka's desert tortoise; Murphy et al. 2011) differ in distribution, morphology, seasonal activity, reproductive ecology, habitat selection, and genetics (Lamb et al. 1989; McLuckie et al. 1999; Berry et al. 2002). The divergence between the two desert tortoise species appears to be a classic example of allopatric speciation resulting from geographic isolation 4–8 million years ago (mya) by the Bouse embayment, which is now occupied by the Colorado River (Lamb et al. 1989; Avise et al. 1992; McLuckie et al. 1999; Murphy et al. 2011). *Gopherus agassizii* is distributed primarily in the Mojave Desert and the lower Colorado River subdivision of the Sonoran Desert, west and north of the Colorado River (Berry et al. 2002). *Gopherus morafkai* is found entirely east and south of the Colorado River in the Sonoran Desert region. The general consensus is that the Colorado River has been flowing through continuously along the Arizona–California border for at least 4.3 mya (House et al. 2005; Wilson and Pitts 2010). This waterway resulted in the divergence of multiple species found on either side of the Colorado River (Wood et al. 2013).

Preliminary genetic work identified a possible population of *G. agassizii* east of the Colorado River in the Black Mountains of Arizona (Glenn et al. 1990) and then McLuckie et al. (1999) characterized the population as Mojavean based on mitochondrial DNA and morphometrics. This isolated population of *G. agassizii* has been hypothesized to have resulted from meandering or drying of the river or even as a result of historic or prehistoric human translocation (McLuckie et al. 1999). The Black Mountains and surrounding area exhibit a complex composition of flora where the Mojave and Sonoran desert ecosystems converge (McLuckie et al. 1999). The Black Mountain population of *G. agassizii* is in proximity to multiple other *G. morafkai* tortoise populations, and McLuckie et al. (1999) observed that tortoises characterized as Mojavean based on mtDNA but residing in the Black Mountains occupied habitat more typical of *G. agassizii* than of neighboring *G. morafkai*. Hybridization between *G. agassizii* and *G. morafkai* has been observed in captivity (Edwards et al. 2010) but has not been studied in a natural setting.

In this study, our objective was to describe the distribution of *G. agassizii* and *G. morafkai* where they come into contact in northwestern Arizona and to investigate the occurrence of hybridization among the parental lineages. We tested the hypothesis that despite an apparent lack of prezygotic reproductive isolating mechanisms, *G. agassizii* and *G. morafkai* maintain independent taxonomic identities based on ecological niche specialization. We employed genetic analyses to explore the extent of hybridization and used habitat suitability models to define the topographic, climatic, and vegetative properties of the contact zone and specific distribution of habitat for each species.

## Materials and Methods

### Sample collection

**Figure 1:**
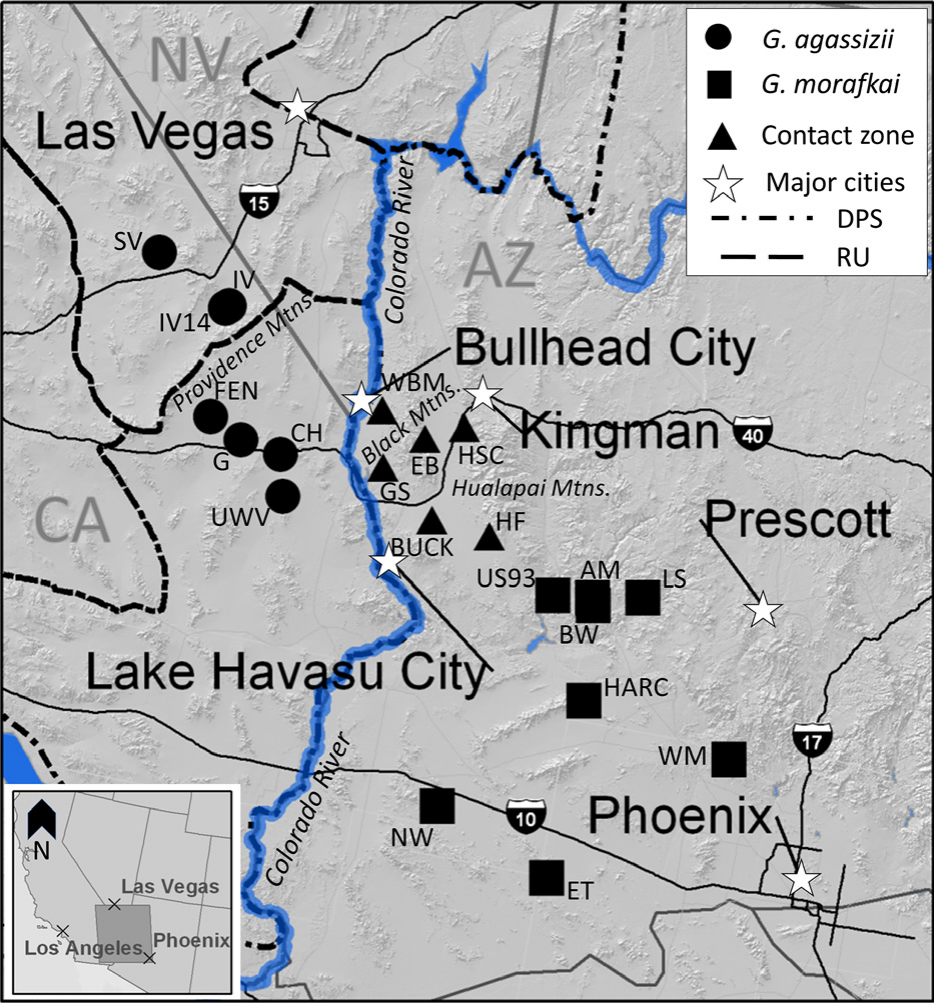
Location of sample sites for *G. agassizii* (circles) in California (CA), *G. morafkai* (squares) in Arizona (AZ), and individuals of undetermined lineage (triangle) in the presumed contact zone (Table1). The California samples represent two different recovery units (RUs); the Colorado Desert RU (FEN, G, CH, and UWV) and the Eastern Mojave RU (SV, IV and IV14), within the distinct population segment (DPS) for *G. agassizii* as defined by the Desert Tortoise Recovery Plan (USFWS 2011). The boundary of the DPS and the state border between California and Arizona is the Colorado River.

We analyzed tissue samples collected from a total of 234 tortoises representing *G. agassizii* in California (*n* - 103), *G. morafkai* in Arizona (*n* - 78), and 53 individuals of undetermined lineage in the presumed contact zone (Table1). The California samples represent two different Federal Recovery Units (RUs; USFWS 2011): the Colorado Desert RU (*n* - 45) and the Eastern Mojave RU which borders the Colorado Desert RU to the west and north but is geographically separated by mountain ranges (Fig.1). Samples from the Colorado Desert RU are west of and across the Colorado River from the Black Mountains. We included Eastern Mojave RU samples in the analysis as an equidistant comparison to Colorado Desert RU sites (Fig.1).

**Table 1:** Sample locality information for a total of 234 desert tortoise samples representing *G. agassizii* in California (*n* - 103), *G. morafkai* in Arizona (*n* - 78), and 53 individuals of undetermined lineage in the secondary contact zone

Location	Sample sites	Site ID	*n*	County	State	Population category
Eastern Mojave RU	Ivanpah	IV	33	San Bernardino	California	*G. agassizii*
	Ivanpah (site 14)	IV14	22	San Bernardino	California	*G. agassizii*
	Shadow Valley	SV	3	San Bernardino	California	*G. agassizii*
Colorado Desert RU	Fenner	FEN	4	San Bernardino	California	*G. agassizii*
	Goffs	G	26	San Bernardino	California	*G. agassizii*
	Chemhuevi	CH	6	San Bernardino	California	*G. agassizii*
	Upper Ward Valley	UWV	9	San Bernardino	California	*G. agassizii*
Black Mountains	South side (near Oatman Hwy)	GS	4	Mohave	Arizona	Contact Zone
	East bajada (long-term monitoring plot)	EB	13	Mohave	Arizona	Contact Zone
	West side (near Silver Creek Rd.)	WBM	18	Mohave	Arizona	Contact Zone
Buck Mountains		BUCK	6	Mohave	Arizona	Contact Zone
Hualapai Mountains	Shingle Canyon	HSC	7	Mohave	Arizona	Contact Zone
	Hualapai Foothills	HF	5	Mohave	Arizona	Contact Zone
Arrastra Mountains		AM	4	Mohave	Arizona	*G. morafkai*
Miller Mountain	Bonanza Wash	BW	5	Yavapai	Arizona	*G. morafkai*
Bismarck Mountain	Little Shipp Wash	LS	10	Yavapai	Arizona	*G. morafkai*
US Highway 93	Between Wickenburg and Wikieup, AZ	US93	10	Yavapai	Arizona	*G. morafkai*
Eagletail Mountains		ET	14	Maricopa	Arizona	*G. morafkai*
Harcuvar Mountains		HARC	16	Yavapai	Arizona	*G. morafkai*
New Water Mountains		NW	4	La Paz	Arizona	*G. morafkai*
Wickenburg Mountains		WM	15	Yavapai	Arizona	*G. morafkai*

We captured desert tortoises by hand following federal and Arizona state protocols (Averill-Murray 2000; Berry and Christopher 2001), collected <1 mL whole blood via brachial, jugular, or subcarapacial venipuncture and stored the samples in 95% EtOH, EDTA, or lithium heparin. The University of Arizona Institutional Care and Use Committee (IACUC) approved all tortoise-handling protocols (IACUC Control nos. 09-138 and 02-120). All California samples were collected between 1990 and 2002 and were previously analyzed by Murphy et al. (2007). Arizona samples were collected between 2005 and 2010. Because tortoises are long-lived animals with long generation times (*ca*. 25 years; USFWS 1994), we made the assumption that major changes in population genetic structure did not occur within the sampling period.

### Sample habitat

Tortoises in the Eastern Mojave RU typically occur on flats, valley bottoms, alluvial fans, and bajadas characterized by a broad range of Mojave Desert vegetation associations (Berry et al. 2002; USFWS 2011). Tortoises from the northern portion of the Colorado Desert RU occur in similar topography and vegetation associations (USFWS 2011). Tortoises in the Black, Buck, and New Water mountains in extreme western Arizona typically occur in habitat similar to that of the Eastern Mojave RU, but can also be found in deeply incised washes. As their distribution moves south and east, tortoises in Arizona will occasionally use flats, valley bottoms, and alluvial fans, but more typically occur on rocky, steep, boulder-strewn slopes and bajadas, and ridges interspersed among shallow to deeply incised washes (Averill-Murray et al. 2002; Riedle et al. 2008; Grandmaison et al. 2010). Tortoise habitat in Arizona comprises an amalgamation of distinct vegetative communities. From west to east, these communities shift from crucifixion-thorn series, pinyon juniper, and Joshua tree series in the Hualapai Foothills, into the paloverde mixed-cacti series with influence of interior chaparral and Mojave desert scrub in the Arrastra Mountains, Bonanza Wash, and Little Shipp Wash (Turner and Brown 1982). At sites in central Arizona (Harcuvar and Wickenburg mountains), tortoises occupy habitat typical of the Arizona Upland vegetative community (Turner and Brown 1982). Sites in the southwest of Arizona (New Water and Eagletail mountains) occur in the lower Colorado River Valley subdivision of the Sonoran Desert interspersed with elements of Arizona Upland vegetation (Turner and Brown 1982).

### Molecular techniques

We isolated genomic DNA from whole blood, salvaged red blood cells, or lymphatic fluid. We amplified an ∼1500-base pair (bp) portion of mitochondrial DNA (including the ND3, arginine tRNA, ND4L, and part of ND4 genes) following methods in Edwards (2003) and Murphy et al. (2007) for PCR conditions. We submitted PCR products to the University of Arizona Genetics Core for DNA sequencing on a 3730XL DNA Analyzer (Applied Biosystems, Carlsbad, CA). We aligned a 1109-bp sequence using CLC DNA Workbench ver. 5.7.1 (CLC Bio, Aarhus, Denmark).

We characterized all samples for 25 loci previously described as short tandem repeats (STRs): Cm58 (Fitzsimmons et al. 1995); Goag03, Goag04, Goag05, Goag06, Goag07, Goag32 (Edwards et al. 2003); Test56 (Hauswaldt and Glenn 2005); GP15, GP19, GP30, GP55, GP61, GP81, GP96, GP102 (Schwartz et al. 2003); ROM01, ROM02, ROM03, ROM04, ROM05, ROM07, ROM10 (Edwards et al. 2011); and ROM08, ROM09 (Davy et al. 2011). We followed protocols in Edwards et al. (2003), Murphy et al. (2007), and Edwards et al. (2011) for PCR conditions and multiplexing. We analyzed electropherograms using GeneMarker 1.85 (SoftGenetics, State College, PA). We used a novel scoring nomenclature for locus Goag05. This locus exhibits a fixed motif in *G. agassizii*, whereas in *G. morafkai* it is observed with both the fixed motif and a functional microsatellite motif (Engstrom et al. 2007). We therefore treated Goag05 as two independent loci, Goag05a and Goag05b where “a” represents the variable motif A and “b” represents the fixed motif B (Engstrom et al. 2007). The pattern of amplification was informative to our investigation of hybridization because samples could easily be identified as *G. morafkai* (Goag05a heterozygous)*, G. agassizii* (Goag05a null plus Goag05b fixed), or potentially admixed (Goag05a homozygous plus Goag05b fixed) based on the amplification of this marker alone.

### Multilocus genetic clustering analyses

We used STRUCTURE 2.3.4 (Pritchard et al. 2000) to define populations in our dataset and to identify admixed individuals. We ran a multilocus STR analysis using all 234 tortoises in the study. We used an admixture model with allele frequencies correlated among populations. We tested for *K* - 1–8 with 10 trials per *K*, each run for 500,000 iterations following a burn-in period of 50,000 Metropolis-coupled Markov chains (MCMC). We used STRUCTURE HARVESTER Online (Earl and Vonholdt 2012) to evaluate STRUCTURE and DeltaK to determine the best fit of *K* for the data following Evanno et al. (2005).

We used GENELAND (Guillot et al. 2005) to delineate the location and shape of hybrid zones. GENELAND incorporates geographic coordinates into its Bayesian model-based clustering algorithm and spatially infers genetic discontinuities between populations. GENELAND assumes that input coordinates are planar such as the Universal Transverse Mercator (UTM) coordinate system. Our sampling locations fall into two different transverse Mercator zones: 11S and 12S (datum WGS84). We first normalized our GPS data to geographic latitude and longitude coordinates and then converted all the points to UTM zone 11S using publically available tools (S. Dutch, Univ of Wisconsin; http://www.uwgb.edu/dutchs/UsefulData/HowUseExcel.HTM). We ran GENELAND for the STR dataset only and for the STR dataset combined with mtDNA haplotype information. We performed 20 independent runs testing for *K* - 1–6 populations for 500,000 iterations each, thinning every 500. We took into account the putative presence of the null allele in our model choice and assumed uncorrelated allele frequencies. We used a 1000-m coordinate uncertainty which is greater than the average home range of *G. morafkai* (13.3 ± 14.72 ha; Averill-Murray et al. 2002) but less than the maximum distance an individual tortoise is known to travel (>30 km; Edwards et al. 2004). For the STR only analyses, we also employed the admixture model of Guedj and Guillot (2011).

### Hybrid characterization

We assessed the presence of hybrid individuals in our dataset several ways; first, we made a qualitative guess based on the mtDNA haplotype and the presence/zygosity of locus Goag05a. We then identified potential hybrids using the inferred admixture proportion (Q) from the STRUCTURE analysis. We also used GeneClass 2 version 2.0.h (Piry et al. 2004) for population assignments using STR loci. We formatted our input data files to include known populations of *G. agassizii* and *G. morafkai* individuals and the individuals of unknown genetic origin residing in the contact zone (Table1). In GeneClass 2, we employed four different methods: 2 Bayesian methods (Rannala and Mountain 1997; Baudouin and Lebrun 2001), a frequency-based method (Paetkau et al. 1995), and a probability computation using Monte-Carlo resampling (Paetkau et al. 2004). We set the assignment threshold of scores to 0.001, and for the probability computation, we simulated 10,000 individuals and set the type 1 error (*alpha*) to 0.01. For each of the four methods, we ran the analysis with and without locus Goag05. Finally, we ran NewHybrids (Anderson 2008) to further predict the presence of admixed individuals in the contact zone using STR loci. We used the default genotype frequency classes in NewHybrids to compute the posterior probability that a hybrid individual belongs to a specified hybrid category (F_1_, F_2,_ or backcross). We performed several trial runs to test the effect of changing the allele frequency prior from Jeffreys-like to uniform and for the mixing proportions prior (without major effect on the outcome). We ran the program three times: with and without Goag05a (500,000 iterations each) and once using gene frequency priors with Goag05a (180,000 iterations). We analyzed results after a burn-in of 50,000.

### Population descriptive statistics

After determining the extent of hybridization in our sample set, we removed all probable hybrid individuals and then reassigned previously unknown individuals to either *G. agassizii* or *G. morafkai*. We then compared descriptive statistics among each of four populations: Eastern Mojave RU, Colorado Desert RU, Arizona *G. agassizii,* and Arizona *G. morafkai*. We used ARLEQUIN v.3.11 (Excoffier et al. 2005) to detect significant departures from Hardy–Weinberg expectations (HWE; Guo and Thompson 1992) and assessed population differentiation using analysis of molecular variance (AMOVA), pairwise *F*_ST_ (Weir and Cockerham 1984), and theta(H) (Ohta and Kimura 1973). We also used ARLEQUIN to test for linkage disequilibrium (nonrandom association between loci) among all pairs of loci within each population (Slatkin and Excoffier 1996). We used FSTAT v.2.9.3.2 (Goudet 1995) to estimate inbreeding coefficients (F_IS_). We used default parameters in FSTAT and ARLEQUIN for all Markov chain tests and permutations. We used BOTTLENECK (Piry et al. 1999) to test for evidence of historical changes in effective population sizes and deviations from equilibrium conditions. We ran 100,000 replications for each of three tests, sign test, standardized difference test, and Wilcoxon test, under I.A.M., T.P.M., and S.M.M. models.

### Coalescent analyses

We performed phylogenetic analyses on the mtDNA sequence data to establish relationships among maternal lineages. We used BEAST v1.7.5 (Drummond and Rambaut 2007) to produce a phylogenetic tree and to establish estimates of time to most recent common ancestor (TMRCA). Previous estimates of mtDNA divergence time between *G. agassizii* and *G. morafkai* have been fairly consistent at 5–6 mya (Avise et al. 1992; Lamb and Lydeard 1994; McLuckie et al. 1999; Edwards 2003). We set a prior of 5.9 ± 0.5 mya, normal probability distribution, for our Bayesian analysis in BEAST based on Edwards (2003) because this estimate used the same mitochondrial locus as in this study. We ran BEAST using haplotype sequences only and included one out-group sequence of *G. berlanderi* (GenBank: DQ649409.1). We selected an HKY substitution model with the gamma parameter set to 4 using MrModeltest 2.3 (Nylander 2004), and we chose a relaxed, log-normal clock and Yule process model. We ran the MCMC for 500,000,000 generations, sampling every 5000 with a burn-in of 10%. We viewed results in TRACER: MCMC Trace Analysis Tool version v1.6.0 (Rambaut et al. 2003) and used TreeAnnotator v1.7.5 (Rambaut and Drummond 2002) to select the maximum clade credibility tree which has the highest product of the posterior probability of all its nodes from the BEAST analysis. We used FigTree version 1.4.0 (Rambaut 2006) to visualize the tree. In addition, we used PAUP* v.4.0b10 (Swofford 2002) to reconstruct maternal phylogenies using both likelihood and parsimony optimality criterion searches to generate tree topologies for comparison with the Bayesian analyses executed with BEAST. We performed analyses only with unique haplotypes giving all characters equal weight and we defined *G. berlandieri* as the out-group. We performed a heuristic search with 100,000 random addition replicates. We estimated support for inferred relationships by conducting 10,000 nonparametric bootstrap replicates. We performed maximum-likelihood analyses using the HKY model of nucleotide evolution.

We used IMa2 version 2.0 (Hey 2010) to estimate gene flow and divergence times among populations. This program estimates the posterior densities of the time of divergence, population size, and gene flow using an MCMC. The model assumes constant population sizes and gene exchange rates that these data inevitably violate; however, this modeling exercise was primarily employed to estimate timing of divergence between cross-river *G. agassizii* populations. We used the three *G. agassizii* populations that we defined for generating descriptive statistics and removed all probable hybrids. We defined the population tree by ((0,1):3,2):4 with Colorado Desert RU - 0, Arizona *G. agassizii* - 1, and Eastern Mojave RU - 2 and populations 3 and 4 representing ancestral nodes. The demographic parameters used by IMa2 are scaled by the mutation rate (*μ*; Hey 2010; Nielsen and Wakeley 2001) which the user must provide and generation time to convert estimates to demographic units. For STRs, estimating *μ* posed some challenges. Microsatellite mutation rates range from 10^−6^ to 10^−2^ per generation (Schlotterer 2000), and there are no empirical data available for desert tortoises. We estimated *μ* across all STR loci using the mean *θ* for STRs (*θ* - 1.5: Table2). Using *θ* - 4N_e_*μ*, we assumed an N_e_ of 10,000 (to account for the ancestral population and large geographic area of sampling) and calculated *μ* - 3.75 × 10^−5^ (0.0001–0.00001). For mtDNA, we calibrated the mtDNA mutation rate for the locus based on *μ* - 4.0 × 10^−9^ per base pair per year as per Edwards (2003). We ran IMa2 using 22 STRs that fit the program criteria plus mtDNA data. We made several initial runs to assess whether the priors and heating conditions were appropriate for the dataset. We experimented with a range of independent heated chains to ensure adequate MCMC mixing (Geyer 1991). We assessed plot trend lines to ensure adequate mixing of MCMC chains and viewed marginal distributions to determine appropriate parameter settings. In our chosen model parameters for IMa2, we set the upper limit of divergence to *t* - 2, population size to *q* - 1, and migration to *m* - 1. We employed a geometric model of Metropolis coupled with 100 independent heated chains and chain heating values of 0.999 to 0.3. Ultimately, we performed four independent runs of 800,000 genealogies each (sampling every 100 steps) after an initial burn-in of 80,000 steps and with each run starting at a different seed value. Replicated analyses converged on the same posterior probabilities. We combined genealogies from all four runs for analysis for a total of 32,000 sampled genealogies. We achieved informative posterior probabilities for mean times of divergence among populations, and we converted the divergence time parameter (t) into years by dividing the geometric mean of the estimated STR and mtDNA mutation rates (3.42 × 10^−5^) and multiplying the generation time.

**Table 2:** Descriptive statistics of 26 STR loci for 4 desert tortoise populations: Eastern Mojave RU (*G. agassizii)*, Colorado Desert RU (*G. agassizii)*, Arizona *G. agassizii,* and Arizona *G. morafkai* with all probable hybrid individuals removed from the dataset. *n* - number of individuals genotyped; % pairwise linkage - linkage disequilibrium (nonrandom association between loci) calculated among all pairs of loci within each population (Slatkin and Excoffier 1996); theta(H) population parameter where *θ - 4N*_*e*_*μ* for diploids (Ohta and Kimura 1973); *H*_obs_ - observed heterozygosity; *H*_exp_ - expected heterozygosity; SD - standard deviation of randomization tests for Hardy–Weinberg equilibrium; and *F*_IS_ - inbreeding coefficient (Weir and Cockerham 1984)

	Eastern Mojave RU	Colorado Desert RU	*G. agassizii* (AZ)	*G. morafkai* (AZ)	Total
*n*	58	45	32	80	214
Theta(H)	1.500	1.500	1.502	1.547	1.512
No. of alleles	7.615	8.192	7.423	8.769	15.115
SD	6.494	7.441	6.307	7.881	12.087
Allelic Range	16.714	15.524	16.091	17.708	19.846
SD	11.718	13.299	13.494	14.505	15.712
% pairwise linkage	4.64	3.48	17.39	17.10	
*H*_obs_	0.568	0.594	0.542	0.539	
SD	0.282	0.265	0.323	0.262	
*H*_exp_	0.627	0.617	0.591	0.623	
SD	0.275	0.270	0.329	0.247	
*F*_IS_ per population	0.095	0.038	0.085	0.137	
*P*-value	0.001	0.016	0.001	0.001	

### Habitat suitability modeling

We tested hypotheses about distribution and habitat use between *G. agassizii* and *G. morafkai* in a region where they co-occur using habitat suitability models and contributing habitat variables for both species represented in a GIS at a 1-km cell resolution. This resolution adequately represents desert tortoise habitat at regional scales (Nussear et al. 2009) and was used to derive topographic (topographic position index [TPI], surface roughness [SRF], surface texture [ST], and solar insolation [SLR]), climatic (summer maximum temperature [TSMX], winter minimum temperature [TWMN], summer/winter temperature difference [TDIFF], winter precipitation [PCPwnt], summer/winter precipitation ratio [PCPrat], and probability of 3 year drought [PCPp3yd]), and vegetative (length of growing season [DUR], photosynthetic activity [AMP], and annual change in photosynthetic activity [AMPt]) variables for the contact zone study area (see Inman et al. 2014 for methods for topographic and climatic variables). We derived vegetation metrics from western CONUS 250 m eMODIS RSP data downloaded from the USGS EROS Center (http://phenology.cr.usgs.gov). We defined the study area by a 150-km buffer of the convex hull around the sampling locations of the 234 sampled tortoises. From the habitat variables, we assigned topographic, climatic, and vegetative values to the sampling location of each animal. Prior to use, we transformed each variable by mean centering to unit variance. We also assigned individuals by genotype class (*G. agassizii*; *G. morafkai*, or admixed) based on the STRUCTURE analysis Q-value and used this to relate habitat use and niche separation to genotype.

Because differences in habitat selection between *G. agassizii* and *G. morafkai* are apparent across their combined ranges, we tested the hypothesis that each genotype occurs on the landscape in different distributions with respect to habitat variables using two-sample Kolmogorov–Smirnov tests. Hypotheses included complete niche separation (as represented by our habitat variables) for the *G. agassizii* and *G. morafkai* genotype classes to niche overlap between admixed and pure individuals. Separately, we tested for habitat selection differences among the different genotype classes by creating a map of two (“Mojave” and “Sonoran”) and three (“Mojave,” “Sonoran,” and “transitional”) landscape designations with respect to the habitat variables using a k-medoids partitioning method (Reynolds et al. 1992) in R (R Core Team 2014) with the package cluster (Maechler et al. 2014). We tested how well sorted the genotype classes are with respect to these landscape designations. If genotype classes follow habitat use patterns similar to their respective species, we hypothesized that the landscape designations derived from habitat variables would geographically separate the genotype classes. We used Cohen's kappa scores (Cohen 1960) for the different genotype classes to test for significance.

Finally, we mapped the contact zone by treating the admixed genotype class as a separate species and using species distribution modeling (SDM; Franklin 2010) to create a probability distribution map of their potential range. We employed the SDM approach because admixed individuals showed overlap with nonadmixed individuals in habitat use and were not easily separated in geographic space because admixed individuals occurred proximal to nonadmixed individuals. We derived estimates of admixed genotype suitability using generalized additive models (GAM) with the package mgcv (version 1.7-6; Wood 2011) in R (version 2.13.1; R Core Development Team 2011) at a spatial resolution of 1 km and considered the 13 habitat variables along with two additional variables: distance to ecotype edge (D2Edge) and distance to Colorado River (D2River). The first, D2Edge, was calculated as the euclidean distance from the interface between the two landscape designations, which were derived from the clustering analyses. We hypothesized that admixed individuals would most likely be located proximal to the boundary between the two landscape designations. We calculated the second layer, D2River, as the euclidean distance from the Colorado River channel, and used this to represent the vicariance between *G. agassizii* and *G. morafkai*. We log-transformed the two distance metrics prior to analyses. Pseudo-absence data were taken as a large random selection (e.g., 10,000) of the study area to characterize areas where hybrids were not present. We evaluated model fit using the area under the receiver operating characteristic curve (AUC; Fielding and Bell 1997; Cumming 2000) in the package ROCR (Sing et al. 2005) and used the Un-Biased Risk Estimator (UBRE; Craven and Wahba 1979) derived from model training data as a measure of model fit. This metric is appropriate for GAM models fit with REML and is analogous to the commonly used AIC metric (Wood 2006).

## Results

### Multilocus genetic clustering analyses

**Figure 2:**
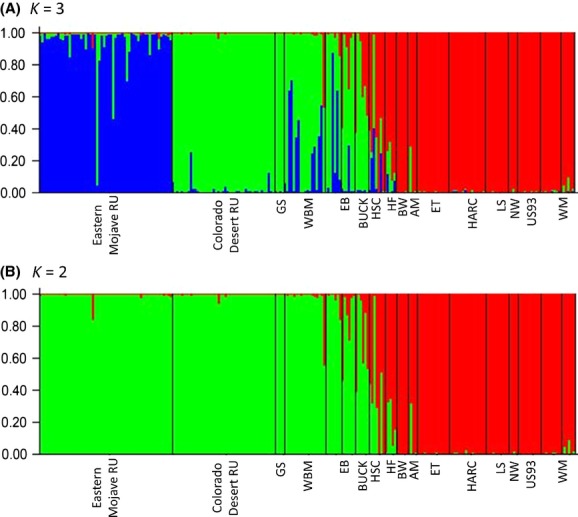
STRUCTRE analysis results for *K* - 3 (A) and *K* - 2 (B) using all 234 tortoises in the study. Site IDs defined in Table1.

**Figure 3:**
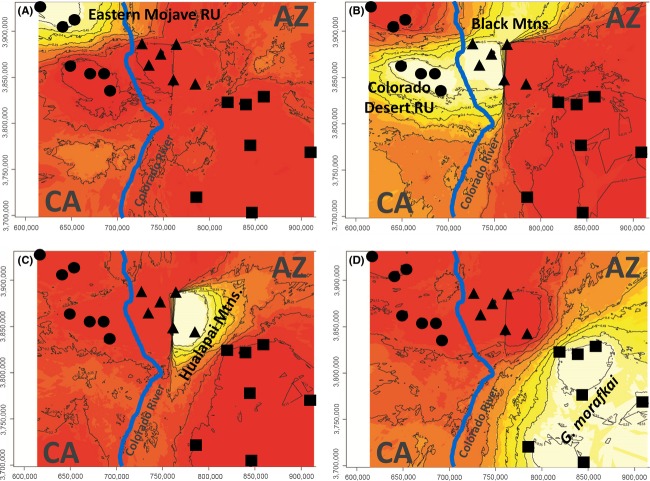
Spatially inferred genetic discontinuities between populations visualized using GENELAND (Guillot et al. 2005). Images generated using STR dataset only. Plotted using UTM planar coordinates, blue line approximates the Colorado River. Cluster A) captures *G. agassizii* sample sites in the Eastern Mojave RU; Cluster B) includes Colorado Desert RU and Black Mountain *G. agassizii* sites in Arizona; Cluster C) includes sites in the Hualapai and Buck mountains, representing the hybrid zone – note the narrow cline between clusters B and C; Cluster D) represents all other *G. morafkai* sites in Arizona.

The STRUCTURE analysis using all 234 tortoises (with and without Goag05) converged on *K* - 2 following Evanno et al. (2005). This differentiation clearly distinguishes the two species, *G. agassizii* and *G. morafkai*. However, examination of the bar plots show that “forcing” *K* - 3 reveals that the 3rd identifiable population is the Eastern Mojave RU and that tortoises in the Colorado Desert RU are more closely affiliated with the Arizona *G. agassizii* population (Fig.2). In the spatial analysis using GENELAND, the optimal number of clusters was *K* - 4, analyzed both with and without mtDNA, based on likelihood estimates (Fig.3). Similar to the STRUCTURE analysis, the Eastern Mojave RU constitutes a genetically distinct population and the Colorado Desert RU and Arizona *G. agassizii* populations cluster together based on posterior probability estimates (Fig.3A,B). In addition, a unique cluster was identified that includes locations in the Hualapai and Buck mountains and this spatially defines the contact zone where hybridization occurs at highest frequencies (Fig.3C).

### Hybrid characterization

Genotype assignments using a combination of STRUCTURE, GENELAND, GeneClass 2, mtDNA haplotype, and NewHybrids show that among our samples in Arizona, purebred *G. agassizii* is restricted to the Black and Buck mountains with a single individual observed in the Hualapai Mountains (Table3). Comparison of the proportion of *G. agassizii* genotypes to *G. morafkai* genotypes is significantly different between the adjacent Black and Hualapai mountains (Table3; two-tailed Fisher's exact test, *P *- 0.001). We identified a total of 19 individuals of apparent mixed ancestry (Tables3 and 4); however, not all individuals were identified by all four of the methods (Table4). Most were identified as F_2_ mixture components based on the NewHybrids analysis. Hybridization does not appear to be biased toward one species or gender (e.g., mtDNA bias). While most hybrid individuals occur within three mountain ranges (Buck, Hualapai, Black), the STRUCTURE and NewHybrids analyses identified one apparent backcrossed individual outside the presumed contact zone in the Arrastra Mountains (Tables3 and 4).

**Table 3:** Genotyping results for 234 desert tortoises based on 26 STR loci. Species identification based primarily on *Q*-values from the STRUCTURE analysis (Pritchard et al. 2000) with additional analyses used to characterize hybridization

Location	Map/Sample Code	*G. agassizii*	*G. morafkai*	Hybrid
Eastern Mojave RU	IV	33		
	IV14	22		
	SV	3		
Colorado Desert RU	FEN	4		
	G	26		
	CH	6		
	UWV	9		
Black Mountains	GS	4		
	EB	9		4
	WBM	16		2
Buck Mountains	BUCK	2		4
Hualapai Mountains	HSC	1	2	4
	HF		1	4
Arrastra Mountains	AM		3	1
Miller Mountain	BW		5	
Bismarck Mountain	LS		10	
US Highway 93	US93		10	
Eagletail Mountains	ET		14	
Harcuvar Mountains	HARC		16	
New Water Mountains	NW		4	
Wickenburg Mountains	WM		15	

**Table 4:** Comparison of analysis methods used to classify 19 individuals as having a possible hybrid origin. Qualitative methods included identity of mtDNA haplotype as either MOJ (*G. agassizii*) or SON (*G. morafkai*) and the presence/zygosity of locus Goag05a where the locus is typically “null” in *G. agassizii,* and thus, an admixed individual is expected to be homozygous at this locus. GeneClass 2 (Piry et al. 2004) was run for four different models and on datasets with and without locus Goag05 (8 total methods); proportion of methods identifying an individual as a hybrid are reported as well as whether the dominant admixture type, *G. agassizii* (Goag) or *G. morafkai* (Gomo), matches that of the maternal lineage. STRUCTURE (Pritchard et al. 2000) was used to classify individuals without prior putative population information. NewHybrids (Anderson 2008) was used to predict specific recombinant classes (F_1_, F_2,_ or backcross)

Location	Site ID	MtDNA Haplotype	Gender	Goag05a	New Hybrids	GeneClass 2		Structure Proportion of admixture (Q)	
						GeneClass/mtDNA mismatch	Proportion of GeneClass2 hybrid detection	Gomo	Goag
Black Mountains	EB	MOJ_A1	M	HOM	F_2_	Yes	7 of 8	0.60	0.40
Black Mountains	EB	SON_01	M	HET	F_2_	Yes	5 of 8	0.37	0.63
Black Mountains	EB	MOJ_A1	M	NULL	F_2_/Bx	No	2 of 8	0.12	0.88
Black Mountains	EB	SON_01	F	NULL	F_2_/Bx	Yes	3 of 8	0.15	0.85
Black Mountains	WBM	MOJ_A01	M	HOM	F_2_	Yes	8 of 8	0.50	0.50
Black Mountains	WBM	MOJ_A01	F	NULL	Pure_Goag	No	2 of 8	0.02	0.98
Buck Mountains	BUCK	SON_05	M	HET	F_2_	No	4 of 8	0.66	0.34
Buck Mountains	BUCK	MOJ_A01	F	NULL	F_2_	No	6 of 8	0.40	0.60
Buck Mountains	BUCK	MOJ_A01	M	HOM	F_2_	No	2 of 8	0.30	0.70
Buck Mountains	BUCK	MOJ_A01	F	HOM	F_2_	Yes	8 of 8	0.54	0.46
Hualapai Mountains	HF	SON_05	M	HET	F_2_	–	0 of 8	0.71	0.29
Hualapai Mountains	HF	SON_01	M	HOM	F_2_	No	1 of 8	0.71	0.29
Hualapai Mountains	HF	MOJ_A01	M	HET	Gomo_Bx	Yes	3 of 8	0.96	0.04
Hualapai Mountains	HF	SON_01	F	HET	Gomo_Bx	–	0 of 8	0.90	0.10
Hualapai Mountains	HSC	MOJ_A01	F	HOM	F_2_	Yes	5 of 8	0.61	0.39
Hualapai Mountains	HSC	SON_01	M	HOM	F_2_	No	2 of 8	0.76	0.25
Hualapai Mountains	HSC	SON_01	F	NULL	F_2_	–	0 of 8	0.63	0.37
Hualapai Mountains	HSC	SON_01	unk.	HOM	F_2_	Yes	6 of 8	0.52	0.48
Arrastra Mountains	AM	SON_01	unk.	HOM	F_2_/Bx	–	0 of 8	0.72	0.28

### Descriptive statistics

Descriptive statistics for the four a priori defined populations (with hybrid individuals removed from analyses) exhibit a remarkable similarity, suggesting shared patterns of demographic history, including population size (*θ*) and allelic richness (Table2). Among the 345 pairwise comparisons of 26 total STR loci, significant linkage disequilibria were detected between 12 and 16 locus pairs in the Eastern Mojave and Colorado Desert RUs, respectively, and for 59 and 60 locus pairs in both the Arizona *G. agassizii* and *G. morafkai* populations (Table2). F_IS_ and deviations from HWE were not consistent across loci and at all sites within the hybrid zone (Table2). Using program BOTTLENECK, all four populations exhibited an excess heterozygosity consistent with a recent reduction in effective population size with significant *P*-values (*P* < 0.05) for all three tests for the T.P.M and S.M.M models, but not the I.A.M model (except Arizona *G. agassizii* was not significant for the Wilcoxon test, T.P.M). The AMOVA confirms the strong differentiation between the two species, *G. agassizii* and *G. morafkai,* as well as the greater similarity of the Colorado Desert RU and Arizona *G. agassizii* populations. Population differentiation (pairwise *F*_ST_) between the Arizona *G. agassizii* population and the Colorado Desert RU and Eastern Mojave RU were 0.013 and 0.051, respectively, and between the two RUs was 0.084. Pairwise *F*_ST_ among *G. morafkai* and the Arizona *G. agassizii,* Colorado Desert RU, and Eastern Mojave RU populations was 0.218, 0.242, and 0.222, respectively.

### Coalescent analyses

**Figure 4:**
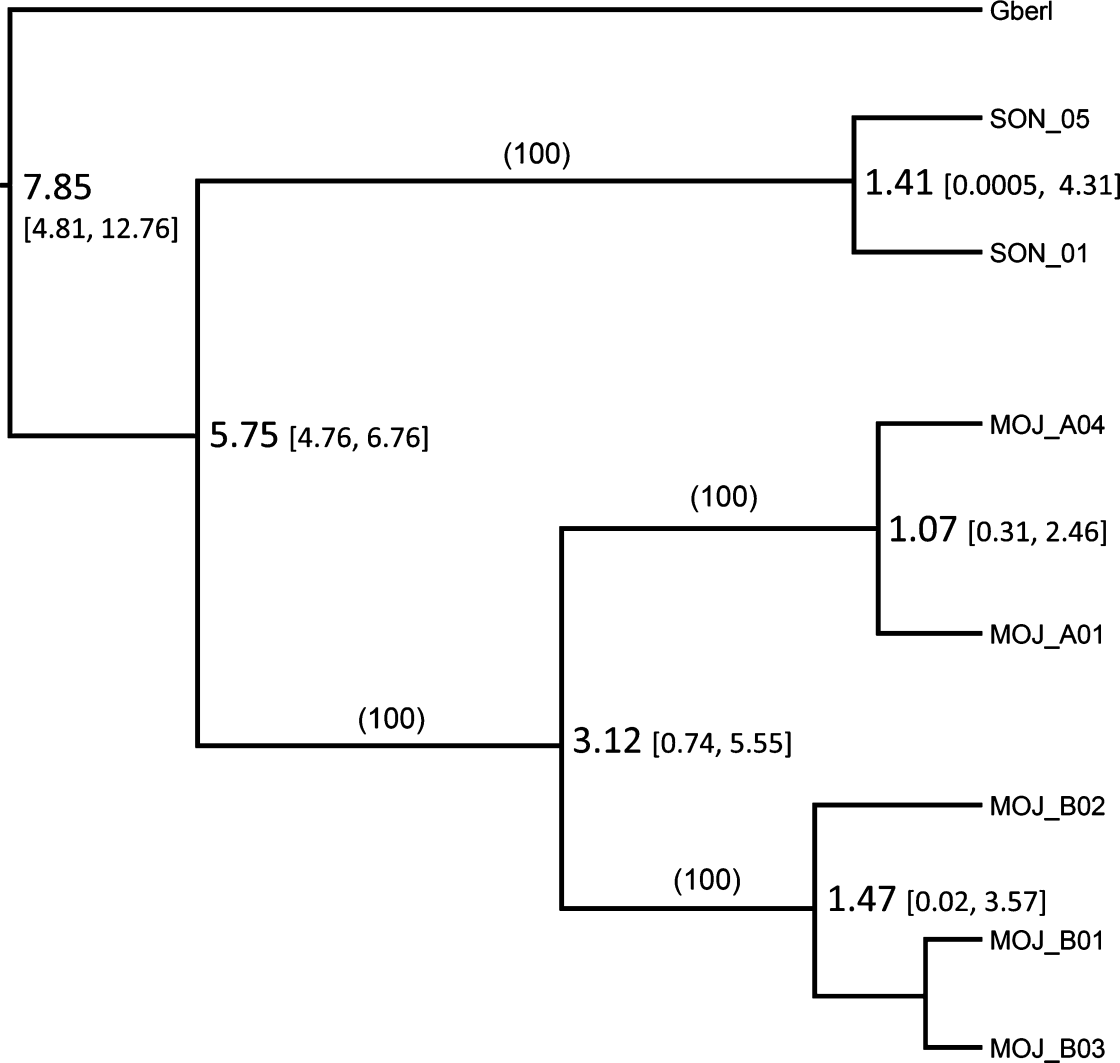
Genealogical reconstruction of mitochondrial haplotypes (ND3, arginine tRNA, ND4L, and part of ND4) rooted with *G. berlandieri* out-group. Nodes labeled with mean TMRCA as million years ago with brackets containing 95% highest posterior density intervals; generated using BEAST v1.7.5 (Drummond and Rambaut 2007). 50% majority-rule consensus tree bootstrap values in parentheses.

We generated mtDNA sequences for 195 samples and identified seven haplotypes, all of which had been previously described (Appendix 1). In Arizona, *G. agassizii* haplotypes were predominant in the Black and Buck mountains and were not found outside of the putative contact zone. The reconstructed gene tree had strong support (Fig.4) and divergence time between *G. agassizii* and *G. morafkai* at this locus was consistent with previous studies, 5.75 mya (Fig.4: Avise et al. 1992; Lamb and Lydeard 1994; McLuckie et al. 1999; Edwards 2003). Both the maximum-likelihood and parsimony reconstructions were consistent with the Bayesian results for all major clades.

The IMa2 analysis estimated the TMRCA between the Colorado Desert RU and Arizona *G. agassizii* populations to be 2408 years. (95% CI; 732–5120) and both of these populations from the Eastern Mojave RU population at 1.38 mya (95% CI; 1.19–1.46). Parameter estimates of population size for the Colorado Desert RU and Arizona *G. agassizii* populations were similar (*q* - 0.426 ± 0.18 and 0.490 ± 0.20 respectively) and both smaller than the Eastern Mojave RU (*q* - 0.761 ± 0.11). All existing populations were estimated to be smaller than ancestral populations (*q*3 - 0.976 ± 0.02 and *q*4 - 0.919 ± c0.08). Migration parameter estimates were similar between the Colorado Desert RU and Arizona *G. agassizii* population (0.426 and 0.490 respectively); however, the distributions of posterior densities for migration parameters did not obtain levels near either the upper or the lower limit of the prior and thus may not be reliable.

### Habitat suitability modeling

**Figure 5:**
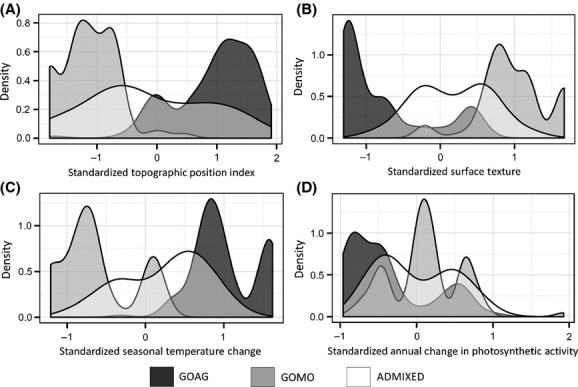
Covariate distributions of genotype class with respect to topographic (A, B), climatic (C), and vegetative (D) habitat variables. Genotype classes characterized as *G. agassizii* (GOAG), *G. morafkai* (GOMO), or admixed. (Not all habitat variables analyzed are presented in figure).

Niche separation among the genotype classes was apparent for most of the habitat variables (Fig.5), with the most drastic differences occurring between *G. agassizii* and *G. morafkai* genotype classes. In general, the covariate distributions for admixed genotype classes overlapped with both the *G. agassizii* and *G. morafkai* genotypes, although their distributions were compressed (Fig.5). All habitat variables showed significant differences between the *G. agassizii* and *G. morafkai* genotypes (Appendix 2), with summer/winter temperature difference (TDIFF) and topographic position index (TPI) showing the highest Kolmogorov–Smirnov statistics. Admixed genotypes were less separated from the other genotypes (Appendix 2). When compared to nonadmixed genotypes, admixed genotypes showed significantly different distributions in multiple habitat layers, with distance to Colorado River (D2River) showing the highest Kolmogorov–Smirnov statistic (Appendix 2).

**Figure 6:**
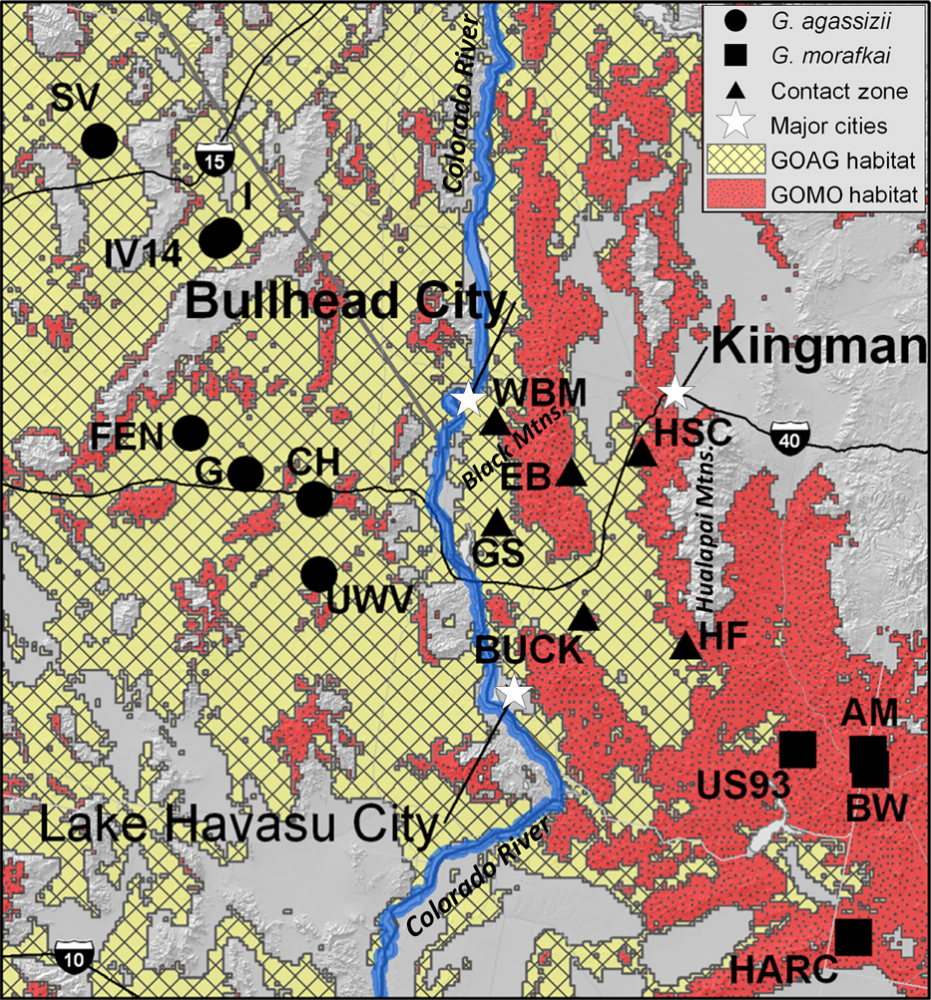
Probability distribution map of the potential range of *G. agassizii* and *G. morafkai* where “stippled pattern” represents predicted *G. morafkai* (Gomo) habitat and “boxed pattern” represents predicted *G. agassizii* (Goag) habitat. Gray background is not predicted to be habitat for desert tortoises. Habitat suitability modeling of habitat use and niche separation of species based on the genotype of sampled individuals (*G. agassizii*; *G. morafkai*, or admixed) determined from the STRUCTURE analysis Q-values. Sampling sites include *G. agassizii* (circles) in California, *G. morafkai* (squares) in Arizona, and individuals from the secondary contact zone in Arizona (triangles). Location is an inset map of Fig.1 and is bisected east/west by the Colorado River.

The two landscape designations resulting from the k-medoids partitioning of the habitat variables accurately predicted the geographic location of the *G. agassizii* and *G. morafkai* genotypes and misclassified only three individuals of the nonadmixed genotypes, resulting in classification that was significantly better than random (*K* - 0.971, *z* - 14.3, *P* < 0.0001). The admixed genotypes were predicted less accurately with three landscape designations, with only 5 of the 17 admixed genotypes identified correctly. Thus, the model with two landscape designations better explains the data than a model with three landscape designations, where admixed individuals were treated as an independent category. The probability distribution map generated from our habitat suitability modeling accurately predicted the locations where we currently observe *G. agassizii* in Arizona as well as the transitional sites where we observe hybridization (Fig.6). The model also predicted areas in Arizona outside of our sampling area that may be suitable for *G. agassizii* (Fig.6). Model performance was generally high, with an AUC score 0.945, far above that of random prediction (Fielding and Bell 1997).

## Discussion

### Biogeography

Our results are largely consistent with the Colorado River having acted as a geographic barrier between *G. agassizi*i and *G. morafkai*, resulting in the deep divergence between these two species (Fig.4). As the actual mutation rates of these loci cannot easily be estimated for these taxa, we rely primarily on the well-established divergence of *G. agassizii* and *G. morafkai* lineage by the vicariant influence of the Bouse inundation which now forms the Colorado River boundary between the species (Avise et al. 1992). However, mtDNA mutation rates based on this geological event are inconsistent with fossil records of divergence among other congeners, and it is expected that the molecular estimates of the time of divergence within this genus are too recent (Avise et al. 1992; Bramble and Hutchison 2014). Until a recalibration of the existing molecular clock is performed using distantly related groups, we treat our projected evolutionary rates as conservative estimates (Fig.4).

The presence of *G. agassizii* east of the Colorado River appears to be relatively recent and has resulted in a zone of secondary contact with its congener, *G. morafkai*. In our analysis, *G. agassizii* and *G. morafkai* were separated on the landscape with respect to all habitat variables, although the admixed genotype class exhibited less separation, with topographic position index (TPI) having the highest explanatory value in the separation between the two species (Appendix 2, Fig.5A). TPI classifies both slope position (i.e., ridge top, hillside, flats) and landform category (i.e., bajada, wash, foothills). This variable is substantially different between the distributions of *G. agassizii* and *G. morafkai*, with *G. agassizii* generally occurring in areas with higher values (corresponding to alluvial fans and valley bottoms), whereas *G. morafkai* tend to occur in areas with lower values (corresponding to foothills, hillside slopes, and more mountainous terrain) (Nussear and Tuberville 2014). The genetic samples reported here are subsamples of a larger geographic area that has yet to be genotyped, yet does not present apparent geographic barriers to tortoise dispersal. In fact, tortoise presence and habitat suitability modeling provide additional areas where the genotype could be either *G. agassizii* or hybrids of *G. agassizii* and *G. morafkai* that are east and south of the Colorado River (Fig.6). Specifically, habitat for *G. agassizii* is predicted to extend further north of the Black Mountains (Fig.6), although there are not many affirmative tortoise localities across Detrital Valley and toward the north end of the White Hills, Arizona (C.A. Jones, pers obs.).

Our IMa2 data analysis suggests a recent shared ancestry (∼2400 years) between *G. agassizii* populations directly across the Colorado River. The Arizona population of *G. agassizii* in the Black Mountains does not constitute a genetically distinct population unto itself but closely resembles the cross-river, Colorado Desert RU population. The IMa2 program makes assumptions on a large number of parameters which our data obviously violated, for example, modeling constant population sizes. This is not a realistic assumption for *G. agassizii* and *G. morafkai* which likely experienced reductions of population size and therefore genetic diversity during the late Pleistocene (Edwards 2003). Despite this, we used IMa2 for modeling general patterns and thus these simplifications were necessary. We interpret our results in light of these biases. The relative proportion of shared mtDNA haplotypes and the absence of novel mtDNA haplotypes in the Arizona *G. agassizii* population also indicate that this small population has not been isolated with enough time for drift or mutation to drive divergence (Avise et al. 1987).

We observed much greater genetic differentiation between the Eastern Mojave RU and the Colorado Desert RU in California than between the Arizona *G. agassizii* populations isolated across the Colorado River. Our results are consistent with Hagerty et al. (2010) in that the Providence and New York mountain ranges are a strong barrier to gene flow between the Ivanpah Valley and the Chemehuevi Valley (Fig.1). This genetic differentiation corresponds to the divergence between the northern Mojave “MOJ_B” maternal haplogroup and the California “MOJ_A” haplogroup which exhibit a deep split between these regions (3.12 mya; Fig.4). The distribution of mtDNA haplogroups despite gene flow with isolation by distance (Murphy et al. 2007; Hagerty and Tracy 2010) suggests a unique population history of northern *G. agassizii* clades. Northern clades potentially diverged during extended periods of isolation from southern populations; however, the current genetic structure may also be maintained by local adaptation.

We considered potential ways that *G. agassizii* might have moved from west to east across the Colorado River: (1) human transport by aboriginal peoples, (2) rafting or floating across the Colorado River, and (3) geological events and movement of the Colorado River channel. The consistency of genetic diversity indices between the Black Mountain population and its adjacent populations in the Colorado RU across the river (Table2) suggest that the hybrid zone was founded by a significant number of individuals. The crossing or isolation of *G. agassizii* on the east side of the river need not have been a single event and may be a result of episodic gene flow. Turtles are susceptible to human-mitigated movements (González-Porter et al. 2011), and desert tortoises in particular are known to have been moved by early humans (Schneider and Everson 1989). For example, the Chemehuevi, a once nomadic people who have occupied the Colorado River basin for thousands of years, were known to use tortoises for food (Schneider and Everson 1989) and treated the tortoises as having an aura of sacredness (Laird 1976), and consequently, might have moved tortoises across the river.

We think that geological events, such as periodic cycles of aggradation, degradation, and avulsion in the lower Colorado River during the Holocene and late Pleistocene, may provide a more likely explanation of the occurrence of *G. agassizii* east of the Colorado River and more closely fall within the estimated timeframe for contact for the two tortoise species. During the last 10,000 years, the lower Colorado River and surrounding areas in the vicinity of the Mojave River Valley have undergone cycles of aggradation and filling of paleovalleys, followed by episodes of degradation, and in some cases stranding of incised river valleys (Howard et al. 2008, 2011). Based on radiocarbon dating of river sediments, this part of the Colorado River was in a narrower and deeper valley 8500 years ago and the valley aggraded and widened between 8500 and 6000 years ago (Howard et al. 2011; pers. comm.). These regional, episodic increases in sediment supply resulted in valley-floor aggradation and incision of the Colorado River, consistent with our timing of *G. agassizii* cross-river shared ancestry. During one of these cycles, habitat with *G. agassizii* may have become stranded as the river narrowed and changed course.

In addition to tortoises, several other species of reptiles, less likely to have been influenced by human transport, exhibit patterns of species differentiation and isolation from one side of the River to another, for example, horned lizards (Mulcahy et al. 2006), rosy boas (Wood et al. 2008), and night snakes (Mulcahy 2008). Other species show cross-river patterns of shared species, haplotypes, or clades; desert iguana and chuckwalla (Lamb et al. 1992), western diamondback rattlesnake (Castoe et al. 2007), spiny lizards (Leache and Mulcahy 2007), fringe-toed sand lizards (Gottscho et al. 2014), and western shovel-nosed snakes (Wood et al. 2014). However, the estimated timing of cross-river dispersal for most of the above species as well as several others (Wood et al. 2013) is greater than the ∼2400 year time span we discuss here for the presence of *G. agassizii* in the Black Mountains.

### Hybridization

Many turtle species are known to hybridize (Lutterschmidt et al. 2007; Vilaca et al. 2012; Parham et al. 2013), and we documented that hybridization naturally occurs between *G. agassizii* and *G. morafkai* at a secondary contact zone in northwestern Arizona. However, we did not always have continuity of results across the different methods we used to detect hybrids (Table4). Bohling et al. (2013) observed that STRUCTURE has the potential to misclassify a pure individual as a hybrid, and Shurtliff et al. (2014) suggest that NewHybrids is much more accurate than STRUCTURE in defining F_1_s, but performs poorly in identifying F_2_s. Nevertheless using multiple methods, we believe we have captured the full breadth of hybridization as is detectable in this dataset. One reason that different methods may not detect hybrids equally is that not all hybrid individuals exhibit the same proportion of admixture. We observed a wide variety of recombinant classes including a high proportion of apparent F_2_s and backcrosses (Table4). This is consistent with the idea that F_1_ individuals may be hard to form under natural conditions and therefore F_2_ and backcrossed individuals should be in greater proportion than F_1_ individuals in a mixed population of two taxa (Arnold 1992, 1997; Reed and Sites 1995). We also observed a disproportionate distribution of hybrid classes, with *G. agassizii* backcrosses dominant in the Black Mountains and *G. morafkai* backcrosses primarily distributed in the Hualapai Mountains (Table4). Bimodal distributions of hybrid individuals associated with some habitat variables may reflect this differentiation in hybrid classes (Fig.5).

Within the area we predefined as the contact zone, we observed a relatively small number of individuals (*n* - 19) of hybrid origin. There appears to be limited distance of penetration across the contact zone from either parental or hybrid genotype class, and the distribution of parentals and hybrid classes is suggestive of exogenous selection and/or assortative mating. Hybrid individuals occur at highest frequency in transitional habitat (Figs.5 and 6), but were not necessarily “bounded” there (Moore 1977). That the pattern of divergence is maintained along transitional habitat is highly suggestive of ecological segregation (Tarroso et al. 2014) and we might assume that interactions between genotype and environment determine the genetic structure of the hybrid population – for example, selection gradient due to environmental heterogeneity (Endler 1977). While highly discriminating between the *G. agassizii* and *G. morafkai*, our topographic, climatic, and vegetative variables could not differentiate as easily admixed individuals from nonadmixed individuals (Appendix 2). Instead, distance to the Colorado River (*D2River*; Appendix 2) provided strong distinction between them and was included in the model used to predict hybrid zones. This variable may be interpreted as the likelihood that *G. agassizii* and *G. morafkai* might come into contact with each other. The results of the k-medoids partitioning support that hybrid individuals do not occupy a unique or novel environment exclusive of parental genotypes. The geographic distribution of hybrid individuals, and thus the hybrid zone, is likely a result of proximity to phylogenetic recontact zones, and not a result of unique habitat selection by hybrids. We therefore propose a geographical selection gradient model (GSGM) consistent with Endler (1977) to explain the distribution of genotypes at this secondary contact zone.

Theoretically, a hybrid zone can remain stable through a balance of dispersal and selection against hybrid genotypes (Barton and Hewitt 1985). The role of effective population size (*N*_e_) is important in interpreting these findings, as the predicted habitat appropriate for *G. agassizii* is limited east of the Colorado River in Arizona (Fig.6). Also, because the population is isolated from the rest of its geographic range, *N*_e_ is expected to be small. With low *N*_e_, genetic drift reduces the effectiveness of selection, and populations with low *N*_e_ may exhibit greater introgression (Nichols and Hewitt 1986). Hybrid individuals can escape selection where they are locally abundant despite having lower fitness, when introgression is constant from outside the zone (Nichols 1989). Although desert tortoises exhibit strong site tenacity, dispersal ability of a tortoise may exceed the ecotone width (>30 km; Edwards et al. 2004).

The presumed allopatric divergence between these two species suggests that opportunities for the evolution of reproductive isolating mechanisms to prevent hybridization are lacking or are minimal. As secondary contact has been relatively recent, there may not have been enough time for this to occur. However, drivers of reproductive isolation such as Haldane's rule (Haldane 1922) do not apply to tortoises as they do not possess heterogametic sex chromosomes and instead have temperature-dependent sex determination. In contrast, mixed genotypes may have functionality in an ecotone, and complete isolation may not always be beneficial for sister taxa (Nei and Nozawa 2011).

### Conservation implications

A distinct population segment (DPS) of *G. agassizii* was federally listed in 1990 as threatened under the U.S. Endangered Species Act based on the conservation status of desert tortoises in this part of their distribution (ESA; USFWS 1990). The DPS was defined as tortoises occurring west and north of the Colorado River (Fig.1; USFWS 1990). The isolated, Arizona population of *G. agassizii* in the Black Mountains and surrounding area is not currently afforded protection under the ESA like its kin across the river because the listed population was geographically delineated; however, it is protected by the Arizona Game and Fish Department (AGFD 2012). Increasing development in this region of Arizona may threaten the viability of this small population of *G. agassizii;* in particular, the proposed realignment of state highways east of Bullhead City would pass directly through primary habitat of this population (ADOT 2014). *Gopherus morafkai* is not federally listed in the USA, but became a candidate for federal listing in 2010 and is considered a Wildlife of Special Concern in Arizona (USFWS 2010, 2012; AGFD 2012).

The identity of species is important for legally determining the lineage of a specimen, as in the case of poaching or designation of conservation easements on private lands (Haig et al. 2004). Unfortunately, *G. agassizii* and *G. morafkai* are challenging to distinguish in the field (Murphy et al. 2011). Where species identification is not easy to determine, geographical delineation may be the best indicator for management (Barrowclough et al. 2011). The existence of natural hybridization between *G. agassizii* and *G. morafkai* further complicates this issue. In the context of species conservation, it is not possible for us to determine which individuals contribute most to the evolutionary potential of the species, or more importantly, which adaptive traits will be most critical in the face of environmental change. For a species to persist, it requires genetic diversity to cope with changes in its environment. With unpredictable stochastic processes, such as climate change, the individuals that have the “best” adaptations may very well be the ones living on edges and in marginal habitats (Eckert et al. 2008; Palstra and Ruzzante 2008; Hardie and Hutchings 2010; Shafer et al. 2011), such as the admixed individuals we observed in this study. Thus, the prudent approach to species conservation is to preserve the entirety of genetic diversity in a species including viable hybrids or populations where the species may benefit from limited introgression. Knowing that the evolutionary potential of a species is directly related to its genetic diversity, we would do best to include the full extent of genetic variation and its maintenance, including the potential for natural hybridization, in conservation efforts.

## Data Archiving

DNA sequences used in this study are available from GenBank:

mtDNA: DQ649394.1, DQ649397.1, DQ649398.1, DQ649399.1, DQ649400.1, DQ649401.1, DQ649405.1, DQ649409.1; STRs: AF546890, AF546895, AF546891, AF546892, AF546889, AF546893, AF546896, AF546894, AF546888, AY317141, AY317142, AY317143, AY317144, AY317145, AY317147, FJ743597, FJ743598, FJ765736, FJ765733, FJ765734, FJ765731, FJ765732, FJ765727, FJ765735, FJ765730, FJ765726, FJ765737, HQ625078, HQ625081, HQ625080, HQ625079.

## Appendix 1

**Table A1:** Distribution of mtDNA haplotypes (as defined by Murphy et al. 2007) among 195 *G. agassizii* and *G. morafkai* samples characterized in three groupings: “*GOAG”* – representing *G. agassizii* sampled from two Recovery Units (RUs) in California; “*Contact Zone”* – samples collected where *G. agassizii* and *G. morafkai* both occur east of the Colorado River in northwestern Arizona; “*GOMO”* – representing *G. morafkai* sampled in western Arizona

Location	Sample sites	MOJ_A01	MOJ_A04	MOJ_B01	MOJ_B02	MOJ_B03	SON_01	SON_05	*n*
		DQ649394.1	DQ649397.1	DQ649398.1	DQ649399.1	DQ649400.1	DQ649401.1	DQ649405.1	
Eastern Mojave RU	IV			13	3	7			23
	IV14			16		3			19
	SV			3					3
Colorado Desert RU	FEN	4							4
	G	19		1					20
	CH	2		1					3
	UWV	4	1						5
Total “GOAG”		29	1	34	3	10			77
Black Mountains	GS	4							4
	EB	9					2		11
	WBM	14		2	1				17
Buck Mountains	BUCK	5						1	6
Hualapai Mountains	HSC	2					4	1	7
	HF	1					2	2	5
Total “Contact Zone”		35		2	1		8	4	50
Arrastra Mountains	AM						4		4
Hualapai Mountains	LS						7	1	8
Miller Mountain	BW						2	3	5
US Highway 93	US93						5	2	7
Eagletail Mountains	ET						13		13
Harcuvar Mountains	HARC						11	2	13
New Water Mountains	NW							3	3
Wickenburg Mountains	WM						13	2	15
Total “GOMO”							55	13	68
Total All		64	1	36	4	10	63	17	195

## Appendix 2

**Table A2:** Results of two-sample Kolmogorov-Smirnov tests used to compare the occurrence/distribution of tortoise genotype classes on the landscape with respect to multiple habitat variables. Genotype class comparisons between *G. agassizii* (GOAG), *G. morafkai* (GOMO) and admixed individuals (AD) and where nonAD represents a combination of non-admixed genotypes GOAG and GOMO. Topographic variables include; topographic position index (TPI), surface roughness (SRF), surface texture (ST) and solar insolation (SLR). Climatic variables include; summer maximum temperature (TSMX), winter minimum temperature (TWMN), summer/winter temperature difference (TDIFF), winter precipitation (PCPwnt), summer/winter precipitation ratio (PCPrat) and probability of 3 year drought (PCPp3yd). Vegetative variables include; length of growing season (DUR), photosynthetic activity (AMP) and annual change in photosynthetic activity (AMPt). In addition, distance to Colorado River (D2River) and distance to ectype edge (D2Edge) were specifically included to predict the potential distribution of admixed individuals. *P*- and *D*- values derived from KS tests

Variable	Genotype class comparison	*P*	*D*	Genotype class comparison	*P*	*D*	Genotype class comparison	*P*	*D*	Genotype class comparison	*P*	*D*
TPI	GOAG × GOMO	0.000	0.956	GOAG × AD	0.000	0.764	AD × GOMO	0.000	0.854	AD × nonAD	0.000	0.582
SRF	GOAG × GOMO	0.000	0.939	GOAG × AD	0.000	0.764	AD × GOMO	0.000	0.831	AD × nonAD	0.003	0.457
ST	GOAG × GOMO	0.000	0.917	GOAG × AD	0.000	0.704	AD × GOMO	0.000	0.661	AD × nonAD	0.003	0.449
SLR	GOAG × GOMO	0.000	0.427	GOAG × AD	0.047	0.352	AD × GOMO	0.071	0.344	AD × nonAD	0.255	0.255
TSMX	GOAG × GOMO	0.000	0.494	GOAG × AD	0.003	0.468	AD × GOMO	0.023	0.397	AD × nonAD	0.114	0.301
TWMN	GOAG × GOMO	0.000	0.475	GOAG × AD	0.037	0.363	AD × GOMO	0.057	0.356	AD × nonAD	0.132	0.294
TDIFF	GOAG × GOMO	0.000	0.980	GOAG × AD	0.000	0.837	AD × GOMO	0.000	0.902	AD × nonAD	0.000	0.638
PCPwnt	GOAG × GOMO	0.000	0.773	GOAG × AD	0.000	0.527	AD × GOMO	0.000	0.574	AD × nonAD	0.023	0.375
PCPrat	GOAG × GOMO	0.000	0.872	GOAG × AD	0.000	0.610	AD × GOMO	0.000	0.657	AD × nonAD	0.004	0.441
PCPp3yd	GOAG × GOMO	0.000	0.870	GOAG × AD	0.000	0.566	AD × GOMO	0.000	0.598	AD × nonAD	0.006	0.431
DUR	GOAG × GOMO	0.000	0.416	GOAG × AD	0.088	0.322	AD × GOMO	0.134	0.310	AD × nonAD	0.332	0.238
AMP	GOAG × GOMO	0.000	0.519	GOAG × AD	0.001	0.522	AD × GOMO	0.018	0.410	AD × nonAD	0.098	0.309
AMPt	GOAG × GOMO	0.001	0.269	GOAG × AD	0.271	0.257	AD × GOMO	0.148	0.304	AD × nonAD	0.715	0.176
d2River	GOAG × GOMO	0.000	0.831	GOAG × AD	0.000	0.556	AD × GOMO	0.000	0.580	AD × nonAD	0.009	0.416
d2Edge	GOAG × GOMO	0.000	0.402	GOAG × AD	0.161	0.289	AD × GOMO	0.139	0.308	AD × nonAD	0.397	0.226

## References

[b1] Anderson EC (2008). Bayesian inference of species hybrids using multilocus dominant genetic markers. Philos. Trans. R. Soc. Lond. B Biol. Sci.

[b2] Arizona Department of Transportation (2014). http://www.azdot.gov/projects/far-west/sr-95-i-40-to-sr-68-tier-1-eis-study/overview.

[b3] Arizona Game and Fish Department (2012). Arizona's state wildlife action plan: 2012–2022.

[b4] Arnold ML (1992). Natural hybridization as an evolutionary process. Annu. Rev. Ecol. Syst.

[b5] Arnold ML (1997). Natural hybridization and evolution.

[b6] Averill-Murray RC (2000). Survey protocol for Sonoran Desert tortoise monitoring plots: reviewed and revised.

[b7] Averill-Murray RC, Martin BE, Bailey SJ, Van Devender TR, Wirt EB (2002). Activity and behavior of the Sonoran Desert tortoise in Arizona. The Sonoran Desert tortoise; natural history, biology and conservation.

[b8] Avise JC, Arnold J, Ball RM, Bermingham E, Lamb T, Neigel JE (1987). Intraspecific phylogeography – the mitochondrial-DNA bridge between population-genetics and systematics. Annu. Rev. Ecol. Syst.

[b9] Avise JC, Bowen BW, Lamb T, Meylan AB, Bermingham E (1992). Mitochondrial-DNA evolution at a turtles pace – evidence for low genetic-variability and reduced microevolutionary rate in the testudines. Mol. Biol. Evol.

[b10] Barrowclough GF, Gutiérrez RJ, Groth JG, Lai JE, Rock DF (2011). The hybrid zone between northern and California spotted owls in the cascade–sierran suture zone. Condor.

[b11] Barton NH, Hewitt GM (1985). Analysis of hybrid zones. Annu. Rev. Ecol. Syst.

[b12] Baudouin L, Lebrun P (2001).

[b13] Berry KH, Christopher MM (2001). Guidelines for the field evaluation of desert tortoise health and disease. J. Wildl. Dis.

[b14] Berry KH, Morafka DJ, Murphy RW (2002). Defining the desert tortoise(s): our first priority for a coherent conservation strategy. Chelonian Conserv. Biol.

[b15] Bohling JH, Adams JR, Waits LP (2013). Evaluating the ability of Bayesian clustering methods to detect hybridization and introgression using an empirical red wolf data set. Mol. Ecol.

[b16] Bramble DM, Rostal DC, McCoy ED, Mushinsky HR, Hutchison JH (2014). Morphology, Taxonomy, and Distribution of North American Tortoises; An Evolutionary Perspective. Biology and conservation of North American tortoises.

[b17] Castoe TA, Spencer CL, Parkinson CL (2007). Phylogeographic structure and historical demography of the western diamondback rattlesnake (*Crotalus atrox*): a perspective on North American desert biogeography. Mol. Phylogenet. Evol.

[b18] Cohen J (1960). A coefficient of agreement for nominal scales. Educ. Psychol. Meas.

[b19] Craven P, Wahba G (1979). Smoothing noisy data with spline functions. Numer. Math.

[b20] Cumming GS (2000). Using between-model comparisons to fine-tune linear models of species ranges. J. Biogeogr.

[b21] Davy CM, Edwards T, Lathrop A, Bratton M, Hagan M, Henen B (2011). Polyandry and multiple paternities in the threatened Agassiz's desert tortoise, *Gopherus agassizii*. Conserv. Genet.

[b22] Drummond AJ, Rambaut A (2007). BEAST: Bayesian evolutionary analysis by sampling trees. BMC Evol. Biol.

[b23] Earl DA, Vonholdt BM (2012). STRUCTURE HARVESTER: a website and program for visualizing STRUCTURE output and implementing the Evanno method. Conserv. Genet. Resour.

[b24] Eckert CG, Samis KE, Lougheed SC (2008). Genetic variation across species' geographical ranges: the central-marginal hypothesis and beyond. Mol. Ecol.

[b25] Edwards T (2003).

[b26] Edwards T, Goldberg CS, Kaplan ME, Schwalbe CR, Swann DE (2003). PCR primers for microsatellite loci in the desert tortoise (*Gopherus agassizii*, Testudinidae). Mol. Ecol. Notes.

[b27] Edwards T, Schwalbe CR, Swann DE, Goldberg CS (2004). Implications of anthropogenic landscape change on inter-population movements of the desert tortoise (*Gopherus agassizii*. Conserv. Genet.

[b28] Edwards T, Jarchow CJ, Jones CA, Bonine KE (2010). Tracing genetic lineages of captive desert tortoises in Arizona. J. Wildl. Manag.

[b29] Edwards T, Lathrop A, Ngo A, Choffe K, Murphy RW (2011). STR/microsatellite primers for the desert tortoise, *Gopherus agassizii*, and its congeners. Conserv. Genet. Resour.

[b30] Endler JA (1977). Geographic variation, speciation, and clines.

[b31] Engstrom TN, Edwards T, Osentoski MF, Shafer HB, FitzSimmons NN, Georges A, Rhodin AGH, Myers EM (2007). A compendium of PCR Primers for mtDNA, Microsatellite, and Other Nuclear Loci for Freshwater Turtles and Tortoises. Defining turtle diversity: proceedings of a workshop on genetics, ethics, and taxonomy of freshwater turtles and tortoises.

[b32] Evanno G, Regnaut S, Goudet J (2005). Detecting the number of clusters of individuals using the software STRUCTURE: a simulation study. Mol. Ecol.

[b33] Excoffier L, Laval G, Schneider S (2005). Arlequin (version 3.0): An integrated software package for population genetics data analysis. Evol. Bioinform.

[b34] Fielding A, Bell J (1997). A review of methods for the assessment of prediction errors in conservation presence/absence models. Environ. Conserv.

[b35] Fitzsimmons NN, Moritz C, Moore SS (1995). Conservation and dynamics of microsatellite loci over 300-million years of marine turtle evolution. Mol. Biol. Evol.

[b36] Franklin J (2010). Mapping species distributions: spatial inference and prediction.

[b37] Geyer CJ (1991). Markov-Chain Monte-Carlo maximum-likelihood. Proceedings of the 23rd Symposium on the Interface. Comp. Sci. Stat.

[b38] Glenn JL, Straight RC, Sites JW (1990). A plasma-protein marker for population genetic-studies of the desert tortoise (*Xerobates-agassizi*. Great Basin Nat.

[b39] González-Porter GP, Hailer F, Flores-Villela O, García-Anleu R, Maldonado JE (2011). Patterns of genetic diversity in the critically endangered Central American river turtle: human influence since the Mayan age?. Conserv. Genet.

[b40] Gottscho AD, Marks SB, Jennings WB (2014). Speciation, population structure, and demographic history of the Mojave fringe-toed lizard (*Uma scoparia*), a species of conservation cocern. Ecol. Evol.

[b41] Goudet J (1995). FSTAT (version 1.2): a computer program to calculate F-statistics. J. Hered.

[b42] Grandmaison DD, Ingraldi MF, Peck FR (2010). Desert tortoise microhabitat selection on the Florence Military Reservation, south-central Arizona. J. Herpetol.

[b43] Guedj B, Guillot G (2011). Estimating the location and shape of hybrid zones. Mol. Ecol. Resour.

[b44] Guillot G, Mortier F, Estoup A (2005). GENELAND: a computer package for landscape genetics. Mol. Ecol. Notes.

[b45] Guo SW, Thompson EA (1992). Performing the exact test of Hardy–Weinberg proportion for multiple alleles. Biometrics.

[b46] Hagerty BE, Tracy CR (2010). Defining population structure for the Mojave desert tortoise. Conserv. Genet.

[b47] Hagerty BE, Nussear KE, Esque TC, Tracy CR (2010). Making molehills out of mountains: landscape genetics of the Mojave desert tortoise. Landsc. Ecol.

[b48] Haig SM, Mullins TD, Forsman ED, Trail PW, Wennerberg L (2004). Genetic identification of Spotted Owls, Barred Owls, and their hybrids: legal implications of hybrid identity. Conserv. Biol.

[b49] Haldane JBS (1922). Sex ratio and unisexual sterility in hybrid animals. J. Genet.

[b50] Hardie DC, Hutchings JA (2010). Evolutionary ecology at the extremes of species' ranges. Environ. Rev.

[b51] Harrison RG (1993). Hybrid zones and the evolutionary process.

[b52] Hauswaldt JS, Glenn TC (2005). Population genetics of the diamondback terrapin (*Malaclemys terrapin*. Mol. Ecol.

[b53] Hewitt GM (1988). Hybrid zones – Natural laboratories for evolutionary studies. Trends Ecol. Evol.

[b54] Hey J (2010). Isolation with migration models for more than two populations. Mol. Biol. Evol.

[b55] House PK, Pearthree PA, Howard KA, Bell JW, Perkins ME, Faulds JE, Dehler CM, Pederson J (2005). Birth of the lower Colorado River — stratigraphic and geomorphic evidence for its inception near the conjunction of Nevada, Arizona, and California. Geological Society of America, USA.

[b56] Howard KA, Lundstrom SC, Malmon DV, Reheis MC, Hershler R, Miller DM, Hook SJ (2008). Age, distribution, and formation of late Cenozoic paleovalleys of the lower Colorado River and their relation to river aggradation and degradation. Late Cenozoic drainage history of the Southwestern Great Basin and lower Colorado River region: geologic and biotic perspectives.

[b57] Howard KA, Malmon DV, McGeehin JP, Beard LS, Karlstrom KE, Young RA, Billingsley GH, Martin P (2011). Holocene aggradation of the lower Colorado River in Mohave Valley, Arizona. Colorado River evolution 2—origin and evolution of the Colorado River system.

[b58] Inman RD, Nussear KE, Esque TC, Vandergast AG, Hathaway SA, Wood DA (2014).

[b59] Laird C (1976). The Chemehuevis.

[b60] Lamb T, Lydeard C (1994). A molecular phylogeny of the gopher tortoises, with comments on familial relationships within the Testudinoidea. Mol. Phylogenet. Evol.

[b61] Lamb T, Avise JC, Gibbons JW (1989). Phylogeographic patterns in mitochondrial-DNA of the desert tortoise (*Xerobates-agassizi*), and evolutionary relationships among the North-American gopher tortoises. Evolution.

[b62] Lamb T, Jones TR, Avise JC (1992). Phylogeographic histories of representative herpetofauna of the southwestern United States – mitochondrial-DNA variation in the desert iguana (*Dipsosaurus-dorsalis*) and the chuckwalla (*Sauromalus-obesus*. J. Evol. Biol.

[b63] Leache AD, Mulcahy DG (2007). Phylogeny, divergence times and species limits of spiny lizards (*Sceloporus magister* species group) in western North American deserts and Baja California. Mol. Ecol.

[b64] Lutterschmidt WI, Escobar SA, Wilson ED (2007). Multivariate analyses of shell morphology of putative hybrid box turtles. Southeast. Nat.

[b65] Maechler M, Rousseeuw P, Struyf A, Hubert M, Hornik K (2014).

[b66] McLuckie AM, Lamb T, Schwalbe CR, McCord RD (1999). Genetic and morphometric assessment of an unusual tortoise (*Gopherus agassizii*) population in the Black Mountains of Arizona. J. Herpetol.

[b67] Moore WS (1977). Evaluation of narrow hybrid zones in vertebrates. Q. Rev. Bio.

[b68] Mulcahy DG (2008). Phylogeography and species boundaries of the western North American Nightsnake (*Hypsiglena torquata*): revisiting the subspecies concept. Mol. Phylogenet. Evol.

[b69] Mulcahy DG, Spaulding AW, Mendelson JR, Brodie ED (2006). Phylogeography of the flat-tailed horned lizard (*Phrynosoma mcallii*) and systematics of the *P. mcallii-platyrhinos* mtDNA complex. Mol. Ecol.

[b70] Murphy RW, Berry KH, Edwards T, McLuckie AM (2007). A genetic assessment of the recovery units for the Mojave population of the desert tortoise, *Gopherus agassizii*. Chelonian Conserv. Biol.

[b71] Murphy RW, Berry KH, Edwards T, Leviton AE, Lathrop A, Riedle JD (2011). The dazed and confused identity of Agassiz's land tortoise, *Gopherus agassizii* (Testudines, Testudinidae) with the description of a new species, and its consequences for conservation. ZooKeys.

[b72] Nei M, Nozawa M (2011). Roles of mutation and selection in speciation: from Hugo de Vries to the modern genomic era. Genome Biol. Evol.

[b73] Nichols RA (1989). The fragmentation of tension zones in sparsely populated areas. Am. Nat.

[b74] Nichols RA, Hewitt GM (1986). Population-structure and the shape of a chromosomal cline between 2 races of *podisma-pedestris* (orthoptera, acrididae). Biol. J. Linn. Soc.

[b75] Nielsen R, Wakeley J (2001). Distinguishing migration from isolation: a Markov chain Monte Carlo approach. Genetics.

[b76] Nussear KE, Rostal DC, McCoy ED, Mushinsky HR, Tuberville TD (2014). Habitat characteristics of North American tortoises. Biology and conservation of North American tortoises.

[b77] Nussear KE, Esque TC, Inman RD, Gass L, Thomas KA, Wallace CSA (2009).

[b78] Nylander JAA (2004). MrModeltest v2. Program distributed by the author.

[b79] Ohta T, Kimura M (1973). Model of mutation appropriate to estimate number of electrophoretically detectable alleles in a finite population. Genet. Res.

[b80] Paetkau D, Calvert W, Stirling I, Strobeck C (1995). Microsatellite analysis of population-structure in Canadian polar bears. Mol. Ecol.

[b81] Paetkau D, Slade R, Burden M, Estoup A (2004). Genetic assignment methods for the direct, real-time estimation of migration rate: a simulation-based exploration of accuracy and power. Mol. Ecol.

[b82] Palstra FP, Ruzzante DE (2008). Genetic estimates of contemporary effective population size: what can they tell us about the importance of genetic stochasticity for wild population persistence?. Mol. Ecol.

[b83] Parham JF, Papenfuss TJ, Dijk PP, Wilson BS, Marte C, Schettino LR (2013). Genetic introgression and hybridization in Antillean freshwater turtles (*Trachemys*) revealed by coalescent analyses of mitochondrial and cloned nuclear markers. Mol. Phylogenet. Evol.

[b84] Piry S, Luikart G, Cornuet JM (1999). BOTTLENECK: A computer program for detecting recent reductions in the effective population size using allele frequency data. J. Hered.

[b85] Piry S, Alapetite A, Cornuet JM, Paetkau D, Baudouin L, Estoup A (2004). GENECLASS2: a software for genetic assignment and first-generation migrant detection. J. Hered.

[b86] Pritchard JK, Stephens M, Donnelly P (2000). Inference of population structure using multilocus genotype data. Genetics.

[b87] R Core Development Team (2011). R-A language and environment for statistical computing.

[b88] R Core Team (2014). R: a language and environment for statistical computing.

[b89] Rambaut A (2006). http://tree.bio.ed.ac.uk/.

[b90] Rambaut A, Drummond AJ (2002). http://beast.bio.ed.ac.uk/.

[b91] Rambaut A, Suchard MA, Xie W, Drummon AJ (2003). http://beast.bio.ed.ac.uk/.

[b92] Rannala B, Mountain JL (1997). Detecting immigration by using multilocus genotypes. Proc. Natl Acad. Sci. USA.

[b93] Reed KM, Sites JW (1995). Female fecundity in a hybrid zone between 2 chromosome races of the Sceloporus-Grammicus complex (Sauria, Phrynosomatidae). Evolution.

[b94] Reynolds A, Richards G, de la Iglesia B, Rayward-Smith V (1992). Clustering rules: a comparison of partitioning and hierarchical clustering algorithms. J. Math Model. Algor.

[b95] Riedle JD, Averill-Murray RC, Lutz CL, Bolen DK (2008). Habitat use by desert tortoises (*Gopherus agassizii*) on alluvial fans in the Sonoran Desert, South-Central Arizona. Copeia.

[b96] Schlotterer C (2000). Evolutionary dynamics of microsatellite DNA. Chromosoma.

[b97] Schneider JS, Everson GD (1989). The desert tortoise (*Xerobates agassizii*) in the prehistory of the southwestern Great Basin and adjacent areas. J. Calif. Gt. Basin Anthropol.

[b98] Schwartz TS, Osentoski M, Lamb T, Karl SA (2003). Microsatellite loci for the North American tortoises (genus *Gopherus*) and their applicability to other turtle species. Mol. Ecol. Notes.

[b99] Shafer ABA, Cote SD, Coltman DW (2011). Hot spots of genetic diversity descended from multiple Pleistocene refugia in an alpine ungulate. Evolution.

[b100] Shurtliff QR, Murphy PJ, Matocq MD (2014). Ecological segregation in a small mammal hybrid zone: habitat-specific mating opportunities and selection against hybrids restrict gene flow on a fine spatial scale. Evolution.

[b101] Sing T, Sander O, Beerenwinkel N, Lengauer T (2005). ROCR: visualizing classifier performance in R. Bioinformatics.

[b102] Slatkin M, Excoffier L (1996). Testing for linkage disequilibrium in genotypic data using the expectation-maximization algorithm. Heredity.

[b103] Smith KL, Hale JM, Kearney MR, Austin JJ, Melville J (2013). Molecular patterns of introgression in a classic hybrid zone between the Australian tree frogs, *Litoria ewingii* and *L. paraewingi*: evidence of a tension zone. Mol. Ecol.

[b104] Swofford DL (2002). PAUP*. Phylogeneitc analysis using parsimony (*and other methods).

[b105] Tarroso P, Pereira RJ, Martinez-Freiria F, Godinho R, Brito JC (2014). Hybridization at an ecotone: ecological and genetic barriers between three Iberian vipers. Mol. Ecol.

[b106] Turner RM, Brown DE, Brown DE (1982). Sonoran desertscrub. Biotic communities of the American Southwest–United States and Mexico.

[b107] U.S. Fish Wildlife Service (1990). Endangered and threatened wildlife and plants: determination of threatened status for the Mojave population of the desert tortoise. Fed. Reg.

[b108] U.S. Fish and Wildlife Service (1994). Desert tortoise (Mojave population) recovery plan.

[b109] U.S. Fish and Wildlife Service (2010). Endangered and threatened wildlife and plants; 12-month finding on a petition to list the Sonoran population of the desert tortoise as endangered or threatened; proposed rule. Fed. Reg.

[b110] U.S. Fish and Wildlife Service (2011). Revised recovery plan for the Mojave population of the desert tortoise (Gopherus agassizii).

[b111] U.S. Fish and Wildlife Service (2012). Listing priority changes in candidates. Fed. Reg.

[b112] Vilaca ST, Vargas SM, Lara-Ruiz P, Molfetti E, Reis EC, Lobo-Hajdu G (2012). Nuclear markers reveal a complex introgression pattern among marine turtle species on the Brazilian coast. Mol. Ecol.

[b113] Weir BS, Cockerham CC (1984). Estimating F-statistics for the analysis of population structure. Evolution.

[b114] Wilson JS, Pitts JP (2010). Illuminating the lack of consensus among descriptions of earth history data in the North American deserts: a resource for biologists. Prog. Phys. Geogr.

[b115] Wood SN (2006). Generalized additive models – An introduction with R.

[b116] Wood SN (2011). Fast stable restricted maximum likelihood and marginal likelihood estimation of semiparametric generalized linear models. J. Roy. Stat. Soc. B.

[b117] Wood DA, Fisher RN, Reeder TW (2008). Novel patterns of historical isolation, dispersal, and secondary contact across Baja California in the Rosy Boa (*Lichanura trivirgata*. Mol. Phylogenet. Evol.

[b118] Wood DA, Vandergast AG, Barr KR, Inman RD, Esque TC, Nussear KE (2013). Comparative phylogeography reveals deep lineages and regional evolutionary hotspots in the Mojave and Sonoran deserts. Divers. Distrib.

[b119] Wood DA, Fisher RN, Vandergast AG (2014). Fuzzy boundaries: color and gene flow patterns among parapatric lineages of the western shovel-nosed snake and taxonomic implication. PLoS ONE.

